# 4-{4-[(*E*)-(2-Hy­droxy­phen­yl)imino­methyl]phen­oxy}benzene-1,2-di­carbo­nitrile

**DOI:** 10.1107/S1600536812003649

**Published:** 2012-02-04

**Authors:** Hülya Tuncer, Ahmet Orhan Görgülü, Tuncer Hökelek

**Affiliations:** a Fırat University, Department of Chemistry, 23169 Elazığ, Turkey; b Hacettepe University, Department of Physics, 06800 Beytepe, Ankara, Turkey

## Abstract

The asymmetric unit of the title compound, C_21_H_13_N_3_O_2_, contains two independent mol­ecules with a similar structure. In one mol­ecule, the central benzene ring is oriented with respect to the terminal benzene rings at 27.23 (7) and 67.96 (7)°; in the other mol­ecule, the corresponding dihedral angles are 12.42 (7) and 64.55 (7)°. In both molecules, there is a short O—H⋯N interaction involving the OH group and the adjacent N atom. In the crystal, there are O—H⋯N hydrogen bonds, and C—H⋯O and N—H⋯O interactions linking the molecules to form a three-dimensional network. π–π stacking between the pyridine and benzene rings and between the benzene rings [centroid–centroid distances = 3.989 (2), 3.705 (2) and 3.607 (2) Å] may further stabilize the structure. A weak C—H⋯π inter­action is present in the crystal.

## Related literature

For the use of phthalonitriles for preparing symmetrically and unsymmetrically substituted phthalocyanine complexes, see: Leznoff & Lever (1996 ▶). For the widespread applications of phthalocyanines in photodynamic therapy, see: Kartal *et al.* (2006 ▶); Tüfekçi *et al.* (2009 ▶). For the fundamental optical and electronic properties of phthalocyanines, see: McKeown (1998 ▶). For related structures, see: Tuncer *et al.* (2012 ▶); Tüfekçi *et al.* (2009 ▶); Yazıcı *et al.* (2009 ▶); Kartal *et al.* (2006 ▶). For bond-length data, see: Allen *et al.* (1987 ▶).
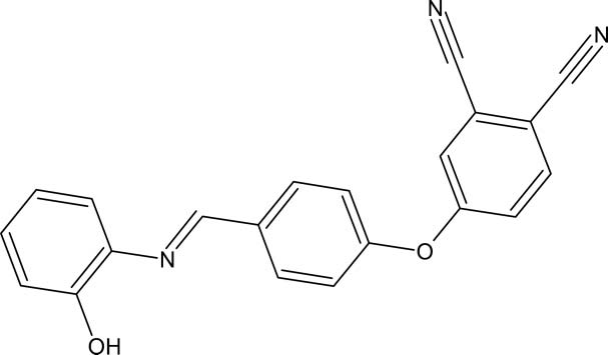


## Experimental

### 

#### Crystal data

C_21_H_13_N_3_O_2_
*M* *_r_* = 339.34
Triclinic, 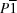

*a* = 9.842 (3) Å
*b* = 13.448 (4) Å
*c* = 14.061 (4) Å
α = 109.940 (15)°
β = 96.937 (16)°
γ = 104.182 (15)°
*V* = 1652.9 (9) Å^3^
*Z* = 4
Mo *K*α radiation
μ = 0.09 mm^−1^
*T* = 100 K
0.40 × 0.23 × 0.13 mm

#### Data collection

Bruker Kappa APEXII CCD area-detector diffractometer
Absorption correction: multi-scan (*SADABS*; Bruker, 2005 ▶) *T*_min_ = 0.975, *T*_max_ = 0.988
28993 measured reflections
8180 independent reflections
4415 reflections with *I* > 2σ(*I*)
*R*_int_ = 0.059

#### Refinement

*R*[*F* ^2^ > 2σ(*F* ^2^)] = 0.052
*wR*(*F* ^2^) = 0.148
*S* = 1.01
8180 reflections
478 parameters
1 restraint
H atoms treated by a mixture of independent and constrained refinement
Δρ_max_ = 0.25 e Å^−3^
Δρ_min_ = −0.26 e Å^−3^

### 

Data collection: *APEX2* (Bruker, 2007 ▶); cell refinement: *SAINT* (Bruker, 2007 ▶); data reduction: *SAINT*; program(s) used to solve structure: *SHELXS97* (Sheldrick, 2008 ▶); program(s) used to refine structure: *SHELXL97* (Sheldrick, 2008 ▶); molecular graphics: *ORTEP-3 for Windows* (Farrugia, 1997 ▶); software used to prepare material for publication: *WinGX* (Farrugia, 1999 ▶) and *PLATON* (Spek, 2009 ▶).

## Supplementary Material

Crystal structure: contains datablock(s) I, global. DOI: 10.1107/S1600536812003649/xu5458sup1.cif

Structure factors: contains datablock(s) I. DOI: 10.1107/S1600536812003649/xu5458Isup2.hkl

Supplementary material file. DOI: 10.1107/S1600536812003649/xu5458Isup3.cml

Additional supplementary materials:  crystallographic information; 3D view; checkCIF report

## Figures and Tables

**Table 1:** Hydrogen-bond geometry (Å, °) *Cg* is the centroid of the C8–C13 ring.

*D*—H⋯*A*	*D*—H	H⋯*A*	*D*⋯*A*	*D*—H⋯*A*
O1—H1⋯N1	0.84	2.24	2.713 (3)	116
O1—H1⋯N2^i^	0.84	2.50	2.993 (3)	119
O1′—H1′⋯N1′	0.84	2.18	2.659 (3)	116
O1′—H1′⋯N3^i^	0.84	2.39	2.948 (3)	125
C3′—H3′⋯N3′^ii^	0.95	2.52	3.344 (4)	145
C5—H5⋯O1′^iii^	0.95	2.40	3.180 (3)	139
C12—H12⋯O1^iv^	0.95	2.35	3.214 (3)	151
C6′—H6′⋯*Cg*^v^	0.95	2.93	3.785 (3)	151

Symmetry codes: (i) 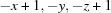; (ii) 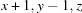; (iii) 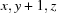; (iv) 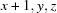; (v) 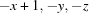.

**Fig. 1.**
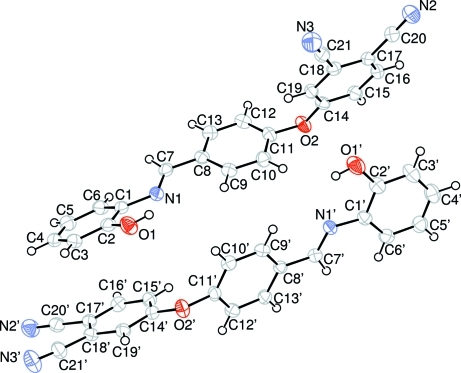
The molecular structure of the title molecule with the atom-numbering scheme. Displacement ellipsoids are drawn at the 50% probability level.

**Fig. 2.**
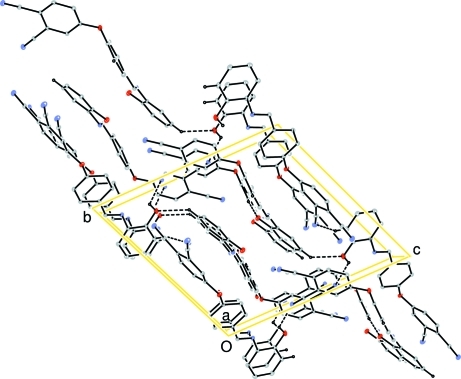
A partial packing diagram. Hydrogen bonds are shown as dashed lines. Hydrogen atoms not involved in hydrogen bonding have been omitted for clarity.

## supplementary crystallographic information

### Comment

Phthalonitriles are used for preparing symmetrically and unsymmetrically substituted phthalocyanine complexes (Leznoff & Lever, 1996). Phthalocyanines have been of great interest to chemists, physicists and industrial scientists. Phthalocyanines have currently been the topic of research because of their wide application fields, such as thin film fabrication, organic pigments, chemical sensors, electrochromic display devices, molecular epitaxic deposition and composites, liquid crystals, photovoltaic cells self-assembled materials. In addition to their extensive use as dyes and pigments, phthalocyanines have found widespread application, in photodynamic therapy (Kartal *et al.*, 2006; Tüfekçi *et al.*, 2009). The fundamental optical and electronic properties of these materials are explained and their potential in non-linear optics, optical data storage, electronic sensors, xerography, solar energy conversion, nuclear chemistry, molecular magnetism, electrochromic displays and heterogeneous catalysis is evaulated by McKeown (1998).

The structures of some phthalonitrile derivatives, C_13_H_8_N_2_O_2_ (Tuncer *et al.*, 2012), C_21_H_12_ClN_3_O (Tüfekçi *et al.*, 2009), C_23_H_17_N_3_O_2_ (Yazıcı *et al.*, 2009) and C_15_H_8_N_2_O_2_ (Kartal *et al.*, 2006) have also been determined.

The asymmetric unit of the title compound, (Fig. 1) contains two crystallographically independent molecules, and the bond lengths are close to standard values (Allen *et al.*, 1987).

The dihedral angles between the hydroxyphenyl rings [A (C1—C6) and A' (C1'—C6')] and the benzene [B (C8—C13), C (C14—C19) and B' (C8'—C13'), C' (C14'—C19')] rings are A/B = 27.23 (7), A/C = 41.88 (7) and A'/B' = 12.42 (7), A'/C' = 73.19 (7) °, while those between the benzene rings B, C and B', C' are B/C = 67.96 (7) and B'/C' = 64.55 (7) °.

In the crystal, intermolecular O—H···N, C—H···O and C—H···N hydrogen bonds (Table 1) link the molecules into a three-dimensional network (Fig. 2), in which they may be effective in the stabilization of the structure. π–π Contacts between the pyridine and benzene rings and between the benzene rings, Cg1—Cg2^i^, Cg1—Cg6 and Cg5—Cg5^ii^ [symmetry codes: (i) 1 - x, 1 - y, 1 - z, (ii) -x, -y, -z, where Cg1, Cg2, Cg5 and Cg6 are the centroids of the rings A (C1—C6), B (C8—C13), B' (C8'—C13') and C' (C14'—C19'), respectively] may further stabilize the structure, with centroid-centroid distances of 3.989 (2), 3.705 (2) and 3.607 (2) Å]. There also exists a weak C—H···π interaction (Table 1).

### Experimental

The title compound has been prepared in two steps. In the first step; 4-hydroxybenzaldehyde (1.86 g, 15.2 mmol) and 4-nitrophthalonitrile (2.64 g, 15.2 mmol) were heated at 353 K in dry DMF (20 ml) with stirring under argon atmosphere. Then, dry fine powdered potassium carbonate (6.00 g, 43.47 mmol) was added in portions (14 × 3.1 mmol) every 10 min. The mixture was heated for a further 18 h. After cooling, the mixture was added into ice-water (200 g). The product was filtered off and washed with NaOH solution (10% w/w) and water until the filtrate was neutral. In the second step; 4-(4-formylphenoxy)benzene-1,2-dicarbonitrile (1.88 g, 7.6 mmol), the product obtained in the first step, and 2-hydroxyaniline (0.83 g, 7.6 mmol) were reacted at 333 K for 4 h in absolute ethanol (50 ml). Recrystallization from absolute ethanol gave a yellow product (yield: 1.55 g, 60%). Single crystals suitable for X-ray diffraction measurement were obtained by slow evaporation of the solution in ethanol (m.p. 433 K).

### Refinement

Atoms H7 and H7' (for CH) were located in a difference Fourier map and were refined by applying restraints. The remaining H-atoms were positioned geometrically with O—H = 0.84 Å for OH H-atoms, and C—H = 0.95 Å for aromatic H-atoms, and constrained to ride on their parent atoms, with *U*_iso_(H) = k × *U*_eq_(C,O), where k = 1.5 for OH H-atoms and k = 1.2 for aromatic H-atoms.

### Crystal data

**Table:**

C_21_H_13_N_3_O_2_	*Z* = 4
*M**_r_* = 339.34	*F*(000) = 704
Triclinic, *P*1	*D*_x_ = 1.364 Mg m^−^^3^
Hall symbol: -P 1	Mo *K*α radiation, λ = 0.71073 Å
*a* = 9.842 (3) Å	Cell parameters from 4598 reflections
*b* = 13.448 (4) Å	θ = 2.3–23.5°
*c* = 14.061 (4) Å	µ = 0.09 mm^−^^1^
α = 109.940 (15)°	*T* = 100 K
β = 96.937 (16)°	Block, yellow
γ = 104.182 (15)°	0.40 × 0.23 × 0.13 mm
*V* = 1652.9 (9) Å^3^	

### Data collection

**Table:**

Bruker Kappa APEXII CCD area-detector diffractometer	8180 independent reflections
Radiation source: fine-focus sealed tube	4415 reflections with *I* > 2σ(*I*)
Graphite monochromator	*R*_int_ = 0.059
φ and ω scans	θ_max_ = 28.5°, θ_min_ = 1.6°
Absorption correction: multi-scan (*SADABS*; Bruker, 2005)	*h* = −12→13
*T*_min_ = 0.975, *T*_max_ = 0.988	*k* = −17→17
28993 measured reflections	*l* = −18→18

### Refinement

**Table:**

Refinement on *F*^2^	Secondary atom site location: difference Fourier map
Least-squares matrix: full	Hydrogen site location: inferred from neighbouring sites
*R*[*F*^2^ > 2σ(*F*^2^)] = 0.052	H atoms treated by a mixture of independent and constrained refinement
*wR*(*F*^2^) = 0.148	*w* = 1/[σ^2^(*F*_o_^2^) + (0.0581*P*)^2^ + 0.3399*P*] where *P* = (*F*_o_^2^ + 2*F*_c_^2^)/3
*S* = 1.01	(Δ/σ)_max_ < 0.001
8180 reflections	Δρ_max_ = 0.25 e Å^−^^3^
478 parameters	Δρ_min_ = −0.26 e Å^−^^3^
1 restraint	Extinction correction: *SHELXL97* (Sheldrick, 2008), Fc^*^=kFc[1+0.001xFc^2^λ^3^/sin(2θ)]^-1/4^
Primary atom site location: structure-invariant direct methods	Extinction coefficient: 0.0067 (11)

### Special details

**Table:**

Geometry. All esds (except the esd in the dihedral angle between two l.s. planes) are estimated using the full covariance matrix. The cell esds are taken into account individually in the estimation of esds in distances, angles and torsion angles; correlations between esds in cell parameters are only used when they are defined by crystal symmetry. An approximate (isotropic) treatment of cell esds is used for estimating esds involving l.s. planes.
Refinement. Refinement of F^2^ against ALL reflections. The weighted R-factor wR and goodness of fit S are based on F^2^, conventional R-factors R are based on F, with F set to zero for negative F^2^. The threshold expression of F^2^ > 2sigma(F^2^) is used only for calculating R-factors(gt) etc. and is not relevant to the choice of reflections for refinement. R-factors based on F^2^ are statistically about twice as large as those based on F, and R- factors based on ALL data will be even larger.

### Fractional atomic coordinates and isotropic or equivalent isotropic displacement parameters (Å^2^)

**Table:**

	*x*	*y*	*z*	*U*_iso_*/*U*_eq_	
O1	0.18465 (16)	0.42838 (13)	0.39648 (13)	0.0415 (4)	
H1	0.2341	0.3847	0.3902	0.062*	
O2	0.81989 (17)	0.09971 (13)	0.27981 (12)	0.0363 (4)	
N1	0.4497 (2)	0.44136 (14)	0.35611 (13)	0.0307 (4)	
N2	0.8274 (2)	−0.23636 (18)	0.53937 (17)	0.0523 (6)	
N3	0.6366 (2)	−0.00505 (17)	0.62696 (17)	0.0470 (6)	
C1	0.3989 (2)	0.53409 (17)	0.36774 (16)	0.0296 (5)	
C2	0.2600 (2)	0.52160 (18)	0.38515 (16)	0.0315 (5)	
C3	0.1965 (3)	0.60425 (19)	0.39298 (17)	0.0342 (6)	
H3	0.1024	0.5953	0.4054	0.041*	
C4	0.2708 (3)	0.69943 (19)	0.38268 (17)	0.0376 (6)	
H4	0.2274	0.7563	0.3878	0.045*	
C5	0.4085 (3)	0.71348 (19)	0.36498 (18)	0.0396 (6)	
H5	0.4592	0.7798	0.3584	0.048*	
C6	0.4720 (3)	0.63074 (18)	0.35691 (17)	0.0351 (6)	
H6	0.5659	0.6400	0.3439	0.042*	
C7	0.5842 (3)	0.45413 (19)	0.37209 (17)	0.0312 (5)	
H7	0.655 (2)	0.5282 (18)	0.3985 (16)	0.028 (6)*	
C8	0.6422 (2)	0.36070 (18)	0.35210 (16)	0.0295 (5)	
C9	0.5531 (2)	0.25160 (18)	0.31442 (17)	0.0338 (5)	
H9	0.4519	0.2370	0.3045	0.041*	
C10	0.6099 (2)	0.16393 (19)	0.29116 (18)	0.0348 (6)	
H10	0.5486	0.0894	0.2639	0.042*	
C11	0.7580 (2)	0.18678 (19)	0.30826 (17)	0.0305 (5)	
C12	0.8491 (2)	0.29320 (19)	0.34658 (17)	0.0339 (5)	
H12	0.9503	0.3073	0.3584	0.041*	
C13	0.7904 (2)	0.37987 (19)	0.36777 (17)	0.0344 (5)	
H13	0.8525	0.4541	0.3936	0.041*	
C14	0.8182 (2)	0.03424 (18)	0.33583 (16)	0.0290 (5)	
C15	0.8887 (2)	−0.04502 (18)	0.30548 (17)	0.0334 (5)	
H15	0.9354	−0.0505	0.2494	0.040*	
C16	0.8912 (2)	−0.11571 (18)	0.35649 (17)	0.0345 (6)	
H16	0.9389	−0.1703	0.3351	0.041*	
C17	0.8244 (2)	−0.10786 (17)	0.43904 (17)	0.0300 (5)	
C18	0.7555 (2)	−0.02651 (17)	0.47002 (16)	0.0285 (5)	
C19	0.7513 (2)	0.04448 (17)	0.41867 (16)	0.0282 (5)	
H19	0.7036	0.0991	0.4397	0.034*	
C20	0.8271 (3)	−0.18017 (19)	0.49414 (19)	0.0373 (6)	
C21	0.6881 (3)	−0.01535 (18)	0.55668 (18)	0.0332 (5)	
O1'	0.48536 (19)	−0.14517 (14)	0.22704 (13)	0.0492 (5)	
H1'	0.4479	−0.0949	0.2288	0.074*	
O2'	−0.00214 (16)	0.24585 (12)	0.13213 (12)	0.0353 (4)	
N1'	0.37253 (18)	−0.08750 (14)	0.07998 (14)	0.0281 (4)	
N2'	0.1000 (2)	0.76703 (18)	0.15817 (17)	0.0486 (6)	
N3'	−0.2416 (2)	0.60508 (18)	0.22270 (19)	0.0549 (6)	
C1'	0.4335 (2)	−0.17435 (18)	0.04730 (17)	0.0304 (5)	
C2'	0.4886 (2)	−0.20314 (19)	0.12727 (18)	0.0354 (6)	
C3'	0.5463 (3)	−0.2899 (2)	0.1072 (2)	0.0429 (6)	
H3'	0.5834	−0.3086	0.1621	0.052*	
C4'	0.5493 (3)	−0.3490 (2)	0.0066 (2)	0.0422 (6)	
H4'	0.5865	−0.4101	−0.0079	0.051*	
C5'	0.4992 (2)	−0.3211 (2)	−0.0738 (2)	0.0402 (6)	
H5'	0.5044	−0.3614	−0.1427	0.048*	
C6'	0.4417 (2)	−0.23440 (19)	−0.05337 (18)	0.0364 (6)	
H6'	0.4071	−0.2153	−0.1087	0.044*	
C7'	0.3167 (2)	−0.05176 (18)	0.01768 (18)	0.0292 (5)	
H7'	0.317 (2)	−0.0807 (17)	−0.0559 (13)	0.040 (7)*	
C8'	0.2428 (2)	0.03159 (17)	0.05018 (16)	0.0263 (5)	
C9'	0.2161 (2)	0.06816 (17)	0.14996 (16)	0.0293 (5)	
H9'	0.2504	0.0410	0.1991	0.035*	
C10'	0.1404 (2)	0.14318 (18)	0.17726 (17)	0.0309 (5)	
H10'	0.1238	0.1690	0.2454	0.037*	
C11'	0.0888 (2)	0.18043 (17)	0.10454 (17)	0.0293 (5)	
C12'	0.1125 (2)	0.14594 (18)	0.00537 (17)	0.0333 (5)	
H12'	0.0766	0.1727	−0.0436	0.040*	
C13'	0.1897 (2)	0.07143 (17)	−0.02080 (17)	0.0310 (5)	
H13'	0.2070	0.0469	−0.0888	0.037*	
C14'	0.0287 (2)	0.34959 (17)	0.12998 (16)	0.0286 (5)	
C15'	0.1547 (2)	0.40575 (18)	0.11284 (17)	0.0321 (5)	
H15'	0.2274	0.3712	0.0987	0.038*	
C16'	0.1749 (2)	0.51264 (18)	0.11636 (17)	0.0326 (5)	
H16'	0.2608	0.5506	0.1030	0.039*	
C17'	0.0709 (2)	0.56471 (17)	0.13924 (16)	0.0294 (5)	
C18'	−0.0559 (2)	0.50719 (18)	0.15786 (16)	0.0291 (5)	
C19'	−0.0774 (2)	0.39984 (18)	0.15198 (16)	0.0295 (5)	
H19'	−0.1644	0.3605	0.1629	0.035*	
C20'	0.0907 (2)	0.6771 (2)	0.14833 (17)	0.0355 (6)	
C21'	−0.1606 (3)	0.56133 (19)	0.19089 (19)	0.0372 (6)	

### Atomic displacement parameters (Å^2^)

**Table:**

	*U*^11^	*U*^22^	*U*^33^	*U*^12^	*U*^13^	*U*^23^
O1	0.0353 (10)	0.0367 (9)	0.0574 (11)	0.0106 (8)	0.0084 (8)	0.0249 (8)
O2	0.0479 (10)	0.0463 (10)	0.0352 (9)	0.0280 (8)	0.0222 (8)	0.0265 (8)
N1	0.0350 (11)	0.0306 (10)	0.0283 (10)	0.0125 (9)	0.0059 (8)	0.0123 (8)
N2	0.0672 (16)	0.0409 (12)	0.0511 (14)	0.0164 (12)	0.0019 (12)	0.0245 (11)
N3	0.0592 (15)	0.0501 (14)	0.0409 (13)	0.0184 (12)	0.0200 (11)	0.0246 (11)
C1	0.0345 (13)	0.0285 (12)	0.0237 (11)	0.0095 (10)	0.0015 (9)	0.0092 (9)
C2	0.0344 (13)	0.0310 (12)	0.0268 (12)	0.0073 (11)	0.0007 (10)	0.0124 (10)
C3	0.0359 (13)	0.0378 (13)	0.0296 (12)	0.0149 (11)	0.0034 (10)	0.0125 (11)
C4	0.0513 (16)	0.0330 (13)	0.0265 (12)	0.0179 (12)	−0.0017 (11)	0.0089 (10)
C5	0.0459 (15)	0.0337 (13)	0.0389 (14)	0.0082 (12)	0.0024 (11)	0.0188 (11)
C6	0.0336 (13)	0.0362 (13)	0.0344 (13)	0.0083 (11)	0.0029 (10)	0.0155 (11)
C7	0.0371 (14)	0.0294 (13)	0.0250 (12)	0.0082 (11)	0.0038 (10)	0.0105 (10)
C8	0.0331 (13)	0.0341 (13)	0.0252 (11)	0.0109 (10)	0.0080 (9)	0.0152 (10)
C9	0.0296 (13)	0.0374 (13)	0.0350 (13)	0.0111 (11)	0.0053 (10)	0.0147 (11)
C10	0.0354 (14)	0.0316 (13)	0.0385 (14)	0.0094 (11)	0.0093 (11)	0.0151 (11)
C11	0.0398 (14)	0.0383 (13)	0.0267 (12)	0.0208 (11)	0.0160 (10)	0.0197 (10)
C12	0.0281 (12)	0.0452 (14)	0.0359 (13)	0.0124 (11)	0.0113 (10)	0.0226 (11)
C13	0.0327 (13)	0.0349 (13)	0.0350 (13)	0.0066 (11)	0.0062 (10)	0.0160 (11)
C14	0.0317 (12)	0.0344 (12)	0.0253 (12)	0.0118 (10)	0.0068 (9)	0.0156 (10)
C15	0.0372 (13)	0.0386 (13)	0.0277 (12)	0.0179 (11)	0.0104 (10)	0.0113 (10)
C16	0.0395 (14)	0.0311 (12)	0.0334 (13)	0.0180 (11)	0.0055 (11)	0.0090 (10)
C17	0.0322 (12)	0.0271 (12)	0.0284 (12)	0.0073 (10)	0.0006 (10)	0.0111 (10)
C18	0.0297 (12)	0.0294 (12)	0.0244 (11)	0.0062 (10)	0.0044 (9)	0.0105 (10)
C19	0.0319 (12)	0.0293 (12)	0.0266 (12)	0.0124 (10)	0.0090 (9)	0.0117 (10)
C20	0.0418 (15)	0.0303 (13)	0.0370 (14)	0.0103 (11)	0.0020 (11)	0.0123 (11)
C21	0.0400 (14)	0.0315 (13)	0.0314 (13)	0.0114 (11)	0.0073 (11)	0.0160 (11)
O1'	0.0714 (13)	0.0581 (11)	0.0373 (10)	0.0373 (10)	0.0189 (9)	0.0272 (9)
O2'	0.0333 (9)	0.0301 (9)	0.0484 (10)	0.0135 (7)	0.0155 (7)	0.0175 (8)
N1'	0.0276 (10)	0.0278 (10)	0.0324 (10)	0.0103 (8)	0.0067 (8)	0.0143 (8)
N2'	0.0527 (14)	0.0440 (13)	0.0553 (14)	0.0165 (11)	0.0085 (11)	0.0265 (11)
N3'	0.0510 (14)	0.0489 (14)	0.0791 (18)	0.0283 (12)	0.0234 (13)	0.0299 (13)
C1'	0.0257 (12)	0.0319 (12)	0.0361 (13)	0.0086 (10)	0.0071 (10)	0.0160 (11)
C2'	0.0382 (14)	0.0411 (14)	0.0343 (14)	0.0165 (12)	0.0145 (11)	0.0184 (11)
C3'	0.0486 (16)	0.0497 (15)	0.0513 (16)	0.0271 (13)	0.0208 (13)	0.0327 (13)
C4'	0.0396 (15)	0.0397 (14)	0.0575 (17)	0.0180 (12)	0.0202 (13)	0.0238 (13)
C5'	0.0368 (14)	0.0396 (14)	0.0420 (15)	0.0158 (12)	0.0111 (11)	0.0094 (12)
C6'	0.0341 (13)	0.0390 (14)	0.0348 (13)	0.0137 (11)	0.0045 (10)	0.0119 (11)
C7'	0.0280 (12)	0.0303 (12)	0.0284 (12)	0.0081 (10)	0.0057 (10)	0.0110 (10)
C8'	0.0235 (11)	0.0253 (11)	0.0279 (12)	0.0052 (9)	0.0026 (9)	0.0104 (9)
C9'	0.0276 (12)	0.0340 (12)	0.0275 (12)	0.0087 (10)	0.0040 (9)	0.0147 (10)
C10'	0.0288 (12)	0.0334 (12)	0.0290 (12)	0.0086 (10)	0.0079 (10)	0.0105 (10)
C11'	0.0253 (12)	0.0253 (11)	0.0356 (13)	0.0088 (10)	0.0067 (10)	0.0088 (10)
C12'	0.0363 (13)	0.0334 (13)	0.0311 (13)	0.0116 (11)	0.0032 (10)	0.0144 (10)
C13'	0.0359 (13)	0.0300 (12)	0.0271 (12)	0.0122 (10)	0.0067 (10)	0.0097 (10)
C14'	0.0308 (12)	0.0263 (12)	0.0274 (12)	0.0090 (10)	0.0034 (9)	0.0095 (9)
C15'	0.0291 (13)	0.0331 (13)	0.0332 (13)	0.0117 (10)	0.0074 (10)	0.0101 (10)
C16'	0.0322 (13)	0.0334 (13)	0.0305 (12)	0.0071 (11)	0.0088 (10)	0.0116 (10)
C17'	0.0338 (13)	0.0309 (12)	0.0246 (11)	0.0107 (10)	0.0049 (9)	0.0119 (10)
C18'	0.0298 (12)	0.0322 (12)	0.0264 (12)	0.0124 (10)	0.0026 (9)	0.0117 (10)
C19'	0.0253 (12)	0.0317 (12)	0.0304 (12)	0.0079 (10)	0.0053 (9)	0.0116 (10)
C20'	0.0397 (14)	0.0377 (14)	0.0321 (13)	0.0133 (11)	0.0052 (10)	0.0171 (11)
C21'	0.0366 (14)	0.0345 (13)	0.0455 (15)	0.0155 (12)	0.0092 (11)	0.0183 (12)

### Geometric parameters (Å, º)

**Table:**

O1—C2	1.362 (3)	O1'—C2'	1.362 (3)
O1—H1	0.8400	O1'—H1'	0.8400
O2—C11	1.411 (2)	O2'—C14'	1.365 (3)
O2—C14	1.366 (2)	O2'—C11'	1.401 (2)
N1—C1	1.420 (3)	N1'—C1'	1.407 (3)
N1—C7	1.274 (3)	N1'—C7'	1.265 (3)
N2—C20	1.142 (3)	C1'—C6'	1.393 (3)
C1—C2	1.398 (3)	C2'—C1'	1.400 (3)
C1—C6	1.390 (3)	C3'—C2'	1.379 (3)
C3—C2	1.382 (3)	C3'—H3'	0.9500
C3—H3	0.9500	C4'—C3'	1.374 (3)
C4—C3	1.374 (3)	C4'—C5'	1.381 (3)
C4—H4	0.9500	C4'—H4'	0.9500
C5—C4	1.387 (3)	C5'—C6'	1.377 (3)
C5—H5	0.9500	C5'—H5'	0.9500
C6—C5	1.383 (3)	C6'—H6'	0.9500
C6—H6	0.9500	C7'—C8'	1.457 (3)
C7—H7	0.98 (2)	C7'—H7'	0.974 (16)
C8—C7	1.461 (3)	C8'—C9'	1.401 (3)
C8—C9	1.389 (3)	C8'—C13'	1.390 (3)
C9—C10	1.383 (3)	C9'—C10'	1.376 (3)
C9—H9	0.9500	C9'—H9'	0.9500
C10—H10	0.9500	C10'—C11'	1.379 (3)
C11—C12	1.368 (3)	C10'—H10'	0.9500
C11—C10	1.386 (3)	C11'—C12'	1.380 (3)
C12—C13	1.384 (3)	C12'—H12'	0.9500
C12—H12	0.9500	C13'—C12'	1.381 (3)
C13—C8	1.393 (3)	C13'—H13'	0.9500
C13—H13	0.9500	C14'—C15'	1.379 (3)
C14—C15	1.387 (3)	C14'—C19'	1.387 (3)
C15—C16	1.375 (3)	C15'—C16'	1.385 (3)
C15—H15	0.9500	C15'—H15'	0.9500
C16—H16	0.9500	C16'—C17'	1.385 (3)
C17—C16	1.388 (3)	C16'—H16'	0.9500
C18—C17	1.398 (3)	C17'—C18'	1.402 (3)
C18—C19	1.384 (3)	C17'—C20'	1.434 (3)
C18—C21	1.437 (3)	C18'—C19'	1.379 (3)
C19—C14	1.389 (3)	C18'—C21'	1.436 (3)
C19—H19	0.9500	C19'—H19'	0.9500
C20—C17	1.438 (3)	C20'—N2'	1.148 (3)
C21—N3	1.147 (3)	C21'—N3'	1.148 (3)
			
C2—O1—H1	109.5	C2'—O1'—H1'	109.5
C14—O2—C11	120.14 (16)	C14'—O2'—C11'	121.23 (17)
C7—N1—C1	120.25 (19)	C7'—N1'—C1'	122.54 (19)
C2—C1—N1	115.60 (19)	C2'—C1'—N1'	114.24 (19)
C6—C1—N1	125.4 (2)	C6'—C1'—N1'	127.7 (2)
C6—C1—C2	118.8 (2)	C6'—C1'—C2'	118.0 (2)
O1—C2—C1	120.78 (19)	O1'—C2'—C1'	119.9 (2)
O1—C2—C3	118.4 (2)	O1'—C2'—C3'	118.8 (2)
C3—C2—C1	120.8 (2)	C3'—C2'—C1'	121.3 (2)
C2—C3—H3	120.3	C2'—C3'—H3'	120.5
C4—C3—C2	119.4 (2)	C4'—C3'—C2'	119.1 (2)
C4—C3—H3	120.3	C4'—C3'—H3'	120.5
C3—C4—C5	120.7 (2)	C3'—C4'—C5'	121.2 (2)
C3—C4—H4	119.6	C3'—C4'—H4'	119.4
C5—C4—H4	119.6	C5'—C4'—H4'	119.4
C4—C5—H5	120.1	C4'—C5'—H5'	120.2
C6—C5—C4	119.9 (2)	C6'—C5'—C4'	119.5 (2)
C6—C5—H5	120.1	C6'—C5'—H5'	120.2
C1—C6—H6	119.9	C1'—C6'—H6'	119.5
C5—C6—C1	120.3 (2)	C5'—C6'—C1'	120.9 (2)
C5—C6—H6	119.9	C5'—C6'—H6'	119.5
N1—C7—C8	122.6 (2)	N1'—C7'—C8'	122.4 (2)
N1—C7—H7	121.4 (12)	N1'—C7'—H7'	121.7 (13)
C8—C7—H7	116.0 (12)	C8'—C7'—H7'	115.8 (13)
C9—C8—C7	121.7 (2)	C9'—C8'—C7'	121.56 (19)
C9—C8—C13	118.4 (2)	C13'—C8'—C7'	119.66 (19)
C13—C8—C7	119.9 (2)	C13'—C8'—C9'	118.66 (19)
C8—C9—H9	119.6	C8'—C9'—H9'	119.8
C10—C9—C8	120.8 (2)	C10'—C9'—C8'	120.5 (2)
C10—C9—H9	119.6	C10'—C9'—H9'	119.8
C9—C10—C11	118.8 (2)	C9'—C10'—C11'	119.2 (2)
C9—C10—H10	120.6	C9'—C10'—H10'	120.4
C11—C10—H10	120.6	C11'—C10'—H10'	120.4
C10—C11—O2	120.6 (2)	C10'—C11'—O2'	116.81 (19)
C12—C11—O2	117.4 (2)	C10'—C11'—C12'	122.0 (2)
C12—C11—C10	121.9 (2)	C12'—C11'—O2'	120.9 (2)
C11—C12—C13	118.5 (2)	C11'—C12'—C13'	118.3 (2)
C11—C12—H12	120.8	C11'—C12'—H12'	120.9
C13—C12—H12	120.8	C13'—C12'—H12'	120.9
C8—C13—H13	119.3	C8'—C13'—H13'	119.3
C12—C13—C8	121.5 (2)	C12'—C13'—C8'	121.4 (2)
C12—C13—H13	119.3	C12'—C13'—H13'	119.3
O2—C14—C15	115.88 (19)	O2'—C14'—C15'	125.50 (19)
O2—C14—C19	123.40 (19)	O2'—C14'—C19'	113.92 (19)
C15—C14—C19	120.7 (2)	C15'—C14'—C19'	120.5 (2)
C14—C15—H15	120.0	C14'—C15'—C16'	119.8 (2)
C16—C15—C14	120.1 (2)	C14'—C15'—H15'	120.1
C16—C15—H15	120.0	C16'—C15'—H15'	120.1
C15—C16—C17	120.4 (2)	C15'—C16'—C17'	120.5 (2)
C15—C16—H16	119.8	C15'—C16'—H16'	119.7
C17—C16—H16	119.8	C17'—C16'—H16'	119.7
C16—C17—C18	119.0 (2)	C16'—C17'—C18'	119.0 (2)
C16—C17—C20	121.3 (2)	C16'—C17'—C20'	122.2 (2)
C18—C17—C20	119.7 (2)	C18'—C17'—C20'	118.8 (2)
C19—C18—C17	121.0 (2)	C17'—C18'—C21'	120.8 (2)
C17—C18—C21	120.0 (2)	C19'—C18'—C17'	120.43 (19)
C19—C18—C21	118.97 (19)	C19'—C18'—C21'	118.6 (2)
C14—C19—H19	120.6	C14'—C19'—H19'	120.2
C18—C19—C14	118.72 (19)	C18'—C19'—C14'	119.6 (2)
C18—C19—H19	120.6	C18'—C19'—H19'	120.2
N2—C20—C17	178.4 (3)	N2'—C20'—C17'	176.1 (3)
N3—C21—C18	178.8 (3)	N3'—C21'—C18'	176.3 (3)
			
C14—O2—C11—C10	−72.6 (3)	C14'—O2'—C11'—C10'	123.8 (2)
C14—O2—C11—C12	111.6 (2)	C14'—O2'—C11'—C12'	−62.8 (3)
C11—O2—C14—C15	−176.49 (19)	C11'—O2'—C14'—C15'	−7.5 (3)
C11—O2—C14—C19	3.3 (3)	C11'—O2'—C14'—C19'	175.44 (18)
C7—N1—C1—C2	−158.2 (2)	C7'—N1'—C1'—C2'	−179.4 (2)
C7—N1—C1—C6	26.0 (3)	C7'—N1'—C1'—C6'	−0.9 (3)
C1—N1—C7—C8	−174.19 (19)	C1'—N1'—C7'—C8'	174.67 (19)
N1—C1—C2—O1	4.0 (3)	N1'—C1'—C6'—C5'	−176.9 (2)
N1—C1—C2—C3	−177.04 (19)	C2'—C1'—C6'—C5'	1.5 (3)
C6—C1—C2—O1	−179.83 (19)	O1'—C2'—C1'—N1'	−2.7 (3)
C6—C1—C2—C3	−0.9 (3)	O1'—C2'—C1'—C6'	178.7 (2)
N1—C1—C6—C5	176.8 (2)	C3'—C2'—C1'—N1'	177.1 (2)
C2—C1—C6—C5	1.0 (3)	C3'—C2'—C1'—C6'	−1.5 (3)
C4—C3—C2—O1	179.47 (19)	C4'—C3'—C2'—O1'	179.7 (2)
C4—C3—C2—C1	0.5 (3)	C4'—C3'—C2'—C1'	−0.1 (4)
C5—C4—C3—C2	−0.2 (3)	C5'—C4'—C3'—C2'	1.7 (4)
C6—C5—C4—C3	0.4 (3)	C3'—C4'—C5'—C6'	−1.7 (4)
C1—C6—C5—C4	−0.8 (3)	C4'—C5'—C6'—C1'	0.1 (4)
C9—C8—C7—N1	−0.2 (3)	N1'—C7'—C8'—C9'	−7.0 (3)
C13—C8—C7—N1	177.8 (2)	N1'—C7'—C8'—C13'	176.9 (2)
C7—C8—C9—C10	176.8 (2)	C7'—C8'—C9'—C10'	−177.02 (19)
C13—C8—C9—C10	−1.2 (3)	C13'—C8'—C9'—C10'	−0.9 (3)
C8—C9—C10—C11	1.4 (3)	C7'—C8'—C13'—C12'	176.5 (2)
O2—C11—C10—C9	−176.21 (19)	C9'—C8'—C13'—C12'	0.3 (3)
C12—C11—C10—C9	−0.5 (3)	C8'—C9'—C10'—C11'	1.2 (3)
O2—C11—C12—C13	175.25 (19)	C9'—C10'—C11'—O2'	172.43 (18)
C10—C11—C12—C13	−0.6 (3)	C9'—C10'—C11'—C12'	−0.9 (3)
C11—C12—C13—C8	0.8 (3)	O2'—C11'—C12'—C13'	−172.77 (19)
C12—C13—C8—C7	−178.0 (2)	C10'—C11'—C12'—C13'	0.3 (3)
C12—C13—C8—C9	0.1 (3)	C8'—C13'—C12'—C11'	0.0 (3)
O2—C14—C15—C16	−179.2 (2)	O2'—C14'—C15'—C16'	−177.6 (2)
C19—C14—C15—C16	1.0 (3)	C19'—C14'—C15'—C16'	−0.8 (3)
C14—C15—C16—C17	−0.5 (3)	O2'—C14'—C19'—C18'	176.48 (18)
C18—C17—C16—C15	−0.5 (3)	C15'—C14'—C19'—C18'	−0.7 (3)
C20—C17—C16—C15	−179.3 (2)	C14'—C15'—C16'—C17'	1.4 (3)
C19—C18—C17—C16	1.0 (3)	C15'—C16'—C17'—C18'	−0.6 (3)
C19—C18—C17—C20	179.9 (2)	C15'—C16'—C17'—C20'	177.1 (2)
C21—C18—C17—C16	−178.7 (2)	C16'—C17'—C18'—C19'	−0.9 (3)
C21—C18—C17—C20	0.1 (3)	C16'—C17'—C18'—C21'	174.4 (2)
C17—C18—C19—C14	−0.6 (3)	C20'—C17'—C18'—C19'	−178.65 (19)
C21—C18—C19—C14	179.2 (2)	C20'—C17'—C18'—C21'	−3.3 (3)
C18—C19—C14—O2	179.77 (19)	C17'—C18'—C19'—C14'	1.6 (3)
C18—C19—C14—C15	−0.4 (3)	C21'—C18'—C19'—C14'	−173.8 (2)

### Hydrogen-bond geometry (Å, º)

Cg is the centroid of the C8-ring.

**Table:**

*D*—H···*A*	*D*—H	H···*A*	*D*···*A*	*D*—H···*A*
O1—H1···N1	0.84	2.24	2.713 (3)	116
O1—H1···N2^i^	0.84	2.50	2.993 (3)	119
O1′—H1′···N1′	0.84	2.18	2.659 (3)	116
O1′—H1′···N3^i^	0.84	2.39	2.948 (3)	125
C3′—H3′···N3′^ii^	0.95	2.52	3.344 (4)	145
C5—H5···O1′^iii^	0.95	2.40	3.180 (3)	139
C12—H12···O1^iv^	0.95	2.35	3.214 (3)	151
C6′—H6′···*Cg*^v^	0.95	2.93	3.785 (3)	151

Symmetry codes: (i) −*x*+1, −*y*, −*z*+1; (ii) *x*+1, *y*−1, *z*; (iii) *x*, *y*+1, *z*; (iv) *x*+1, *y*, *z*; (v) −*x*+1, −*y*, −*z*.
